# Seven chromatin regulators as immune cell infiltration characteristics, potential diagnostic biomarkers and drugs prediction in hepatocellular carcinoma

**DOI:** 10.1038/s41598-023-46107-x

**Published:** 2023-10-30

**Authors:** Jin-wen Chai, Xi-wen Hu, Miao-miao Zhang, Yu-na Dong

**Affiliations:** 1 Department of Oncology, Laizhou Traditional Chinese Medicine Hospital, Laizhou, Shandong China; 2 The First Clinical Medical School, Shandong University of Traditional Chinese Medicine, Jinan, Shandong China; 3 Department of Gastroenterology, Laizhou People’s Hospital, No.1718 Wuli Street, Laizhou, Shandong China

**Keywords:** Cancer genomics, Bioinformatics, Data mining

## Abstract

Treatment is challenging due to the heterogeneity of hepatocellular carcinoma (HCC). Chromatin regulators (CRs) are important in epigenetics and are closely associated with HCC. We obtained HCC-related expression data and relevant clinical data from The Cancer Genome Atlas (TCGA) databases. Then, we crossed the differentially expressed genes (DEGs), immune-related genes and CRs to obtain immune-related chromatin regulators differentially expressed genes (IRCR DEGs). Least absolute shrinkage and selection operator (LASSO) Cox regression analysis was performed to select the prognostic gene and construct a risk model for predicting prognosis in HCC, followed by a correlation analysis of risk scores with clinical characteristics. Finally, we also carried out immune microenvironment analysis and drug sensitivity analysis, the correlation between risk score and clinical characteristics was analyzed. In addition, we carried out immune microenvironment analysis and drug sensitivity analysis. Functional analysis suggested that IRCR DEGs was mainly enriched in chromatin-related biological processes. We identified and validated PPARGC1A, DUSP1, APOBEC3A, AIRE, HDAC11, HMGB2 and APOBEC3B as prognostic biomarkers for the risk model construction. The model was also related to immune cell infiltration, and the expression of CD48, CTLA4, HHLA2, TNFSF9 and TNFSF15 was higher in high-risk group. HCC patients in the high-risk group were more sensitive to Axitinib, Docetaxel, Erlotinib, and Metformin. In this study, we construct a prognostic model of immune-associated chromatin regulators, which provides new ideas and research directions for the accurate treatment of HCC.

## Introduction

Primary liver cancer is the sixth most common primary tumor and the fourth most common cause of cancer-related death worldwide^1^, HCC accounts for 80–90%^2^. Although early HCC can be cured by local ablation, surgical resection or liver transplantation, most HCC cases in the world are in the advanced stage. Targeted systemic therapy and immune checkpoint inhibitors (ICIs) have been proven to be effective treatment options for advanced HCC patients^3,4^, but some patients still do not benefit from them, which may be related to the complex tumor microenvironment (TME). TME is a micro-internal environment with immune inflammatory response, hypoxia and low pH composed of tumor cells and non-tumor cells. These characteristics affect the occurrence and development of tumor and treatment resistance. Among them, immune cells and a variety of factors released by them play an important role in TME^5,6^. Intensive investigation of immune cells and interactions within the TME is important to improve the efficacy of antitumor drugs^7^. Recently, the construction of immune-related risk models using bioinformatics techniques can accurately predict the prognosis of cancer patients and guide the efficacy of ICIs therapy^8,9^.

The heterogeneity of HCC is also related to genetics, epigenetics, proteomics, transcriptomics and metabolomics^10–12^. In recent years, epigenetics has attracted wide attention. Epigenetics does not change the gene sequence to achieve the regulation of gene expression, thus affecting tumorigenesis and all hallmarks of cancer^13–15^. The current studies suggest that epigenetic alterations contribute to promote tumor immune function^16,17^, and epigenetic therapeutics assist in enhancing the effect of immunotherapy^18,19^. For instance, the histone deacetylase inhibitor Belinostat improves the anti-tumor activity of CTLA-4 in a subcutaneous Hepa129 murine HCC model, reflecting the synergistic effect of combined therapy^20^. In HCC animal experiment with the EZH2 inhibitor DZNep and anti-PD-L1 antibody, combination treatment upregulated the expression of Th 1 chemokines and associated tumor antigens, promoted effector T cell infiltration and promoted antitumor immunity^21^. Chromatin regulators (CRs) were vital regulatory element in epigenetics^22^. CRs were mainly classified into three major categories according to roles in epigenetics: DNA methylators, histone modifiers, and chromatin remodelers^23,24^. Mutations in chromatin regulators, such as ARID1A, ARID1B, ARID2, MLL, and MLL3, may contribute to the occurrence and progress of HCC^25^. Chromatin remodeling factor ARID2 expression was negatively correlated with pathological grade and organ metastasis in HCC patients, and ARID2 knockout promotes metastasis in HCC mouse models^26^. Previous studies found that CRs drive epigenetic alterations play an important role in HCC, and also revealed their role patterns in HCC patients^27,28^. The study by Dai et al. identified three CR-related patterns and established the CRs phenotype-related gene signature to predict energy metabolism and cuproptosis activity in HCC^29^. Although the construction of polygenic prognostic models based on CRs and immune-related genes provides potential indicators for the response of ICIs. However, as far as we know, there is no study on the combined analysis of CRs and immune-related genes in HCC.

In this study, we investigated the expression profiles and functional enrichment of immune-related CRs in HCC. We successfully constructed a new prognostic model of HCC based on seven genes, PPARGC1A, DUSP1, APOBEC3A, AIRE, HDAC11, HMGB2, and APOBEC3B. Furthermore, we analyzed the correlation between risk score and clinical characteristics, and explored the correlation between the risk model and the immune microenvironment in HCC. Our results provide a new direction for revealing new biomarkers and new ideas for the accurate treatment of HCC.

## Materials and methods

### Data collection

The RNA sequencing (RNA-seq) data and relevant clinical data of HCC including 374 cancer samples and 50 para-cancer samples were downloaded from The Cancer Genome Atlas (TCGA) database (https://portal.gdc.cancer.gov/)^30^. Another 232 Japanese population HCC samples were obtained from the ICGC portal (https://dcc.icgc.org/projects/LIRI-JP)^31^.

A total of 870 Chromatin regulators (CRs) were retrieved from previous topic research^22^. The lists of immune-related genes were downloaded from the InnateDB (https://www.innatedb.com/) and totaling 1040 human immune-related genes (Supplementary Table 1).

### Identification of immune-related CRs differentially expressed genes (IRCR DEGs)

Based on the genes expression of cancer tissues and para-cancer tissues in the TCGA-HCC dataset, differentially expressed genes (DEGs) were obtained using the “limma” R package according to the criteria of |log2 FC (fold change)|> 1 and adjusted p values < 0.01. Then the “VennDiagram” R package was utilized to obtain IRCR DEGs for the above DEGs. In addition, we obtained the mutations of IRCR DEGs through Gene Set Cancer Analysis (GSCA) (http://bioinfo.life.hust.edu.cn/GSCA/#/)^32^.

### Functional enrichment analyses and gene–gene interaction network

To analyze the identified IRCR DEGs, the Gene Ontology (GO)^33^ and Kyoto Encyclopedia of Genes and Genomes (KEGG)^34^ pathway enrichment analyses were performed and visualized using the “clusterProfiler”^35^ and “GOplot” R package^36^. An adjusted p value < 0.05 was considered the screening criterion for significantly enriched terms.

GeneMANIA^37^, a flexible plugin of Cytoscape, which was applied to identify the genes most relevant to the query gene set and to construct a composite gene–gene functional interaction network.

### Construction of a prognostic model based on IRCRs

We performed lasso-penalized Cox regression analysis to construct the prognostic risk model through glmnet R package. Risk scores were calculated by the following formula:

$${\text{Risk}}\;{\text{score}} = \sum ({\text{coefficient}}_{{\text{i}}} *{\text{expression}}\;{\text{of}}\;{\text{mRNA}}_{{\text{i}}} )$$

All HCC patients were divided into high-risk group and low-risk group by the median risk score. Moreover, we plotted K-M survival curve to evaluate the discrepancy of OS between the two groups by the “Survminer” R package^38^, and time-related receiver operating characteristic (ROC) curves were applied to assess the accuracy of the risk model by the “survivalROC” (version 1.03) packages^39^. The ICGC LIRI-JP dataset was considered as a valid set for further external verification of the prognostic model.

### Construction of nomogram model

We researched the relationship between IRCR-based signature and clinical characteristics in HCC. To verify whether the signature risk score could be used as an independent prognostic factor in HCC patients, univariate and multivariate Cox regression analyses were performed. A nomogram associated with outcome was built to investigate the probability of prognosis for HCC patients. The calibration curve was performed to assess the predictive utility of the nomogram.

### Immune cell infiltration analysis

In order to determine the immune prognostic correlation of IRCR-based signature in HCC, we used CIBERSORT, CIBERSORT-ABS, QUANTISEQ, MCP-counter, XCELL, TIMER, and EPIC algorithms to evaluate the infiltration level of immune cells between high-risk group and low-risk group. Meanwhile, we explored the expression of several immune checkpoints to predict the effect of immune checkpoint blockade therapy. In addition, the TIMER database(https://cistrome.shinyapps.io/timer/)^40^ was used to identify the correlations between 7 IRCRs and six immune cells (B cells, CD4 + T cells, CD8 + T cells, neutrophils, macrophages, and dendritic cells) in HCC.

### Drug sensitivity analysis

The half-maximal inhibitory concentration (IC50) of drugs were analysed by using the Genomics of Drug Sensitivity in Cancer (GDSC, http://www.cancerrxgene.org/)^41^ database, and the drugs sensitivity were predicted by using the “pRRophetic”^42^ R package.

### Statistical methods

R (v.4.0.2) was used for statistical analysis and visualization. Differences between groups were compared using the Wilcoxon rank-sum test. P value < 0.05 was considered statistically significant (ns, p ≥ 0.05; *p < 0.05; **p < 0.01; ***p < 0.001).

### Informed consent

All participating authors give their consent for this work to be published.

## Results

### Identification and mutation analysis of IRCR DEGs in HCC samples

**Figure 1:**
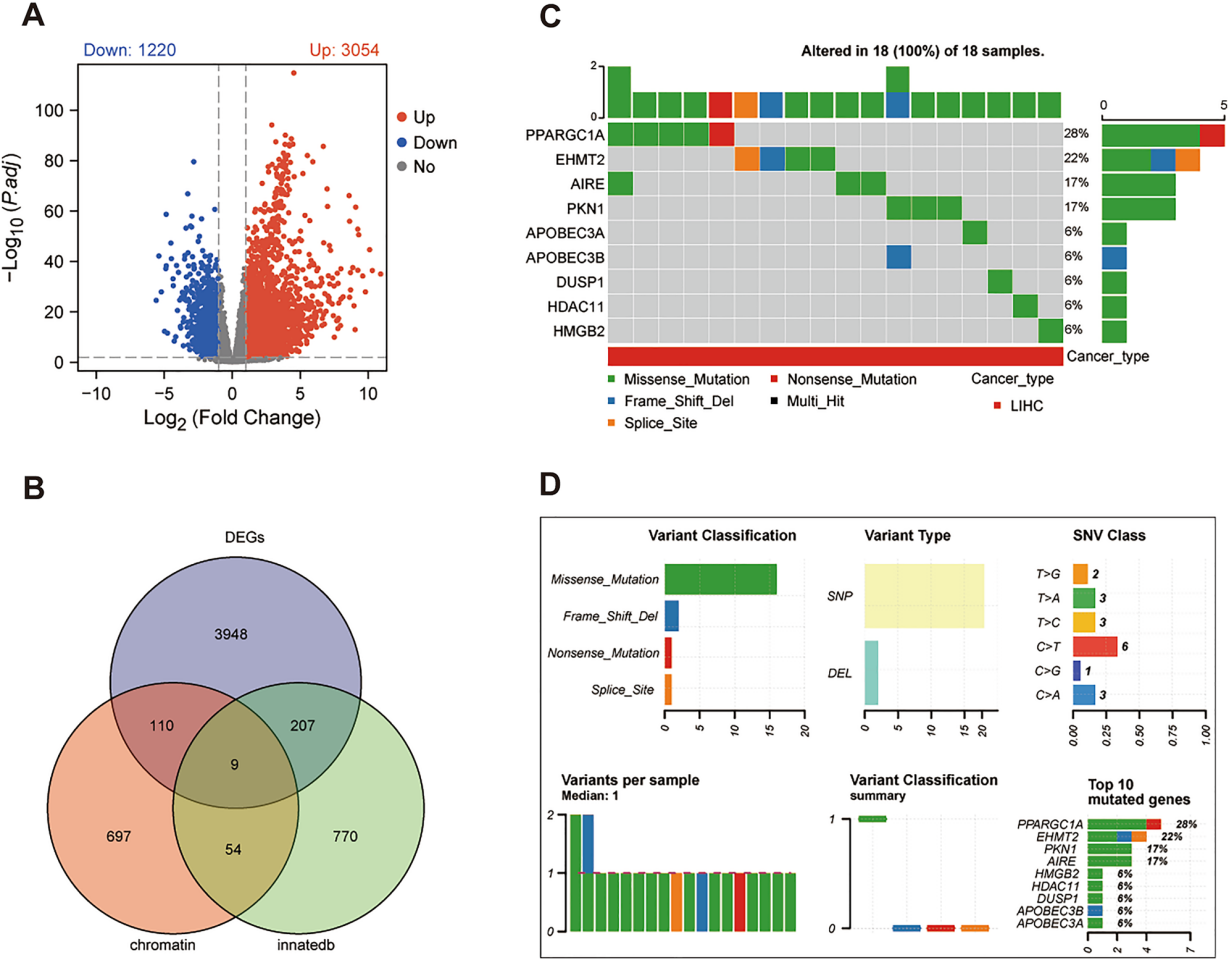
Identification and mutation profile analysis of IRCR DEGs from the TCGA-HCC cohort. (**A**) Volcano plot of 4274 differentially expressed genes. (**B**)The Venn diagram of DEGs, CRs, and immune-related genes were downloaded from the InnateDB. (**C**) Oncoplot displaying the situation of the SNV of IRCR DEGs in HCC samples from TCGA database. (**D**) The SNV classes of IRCR DEGs in TCGA-HCC cohort.

We found 4274 DEGs between the HCC and normal samples, of which 3054 were upregulated and 1220 were downregulated in the HCC samples (Fig. 1A, Supplementary Table 2). 9 IRCR DEGs were obtained by overlapping 4274 DEGs, 870 CRs and 1040 human immune-related genes (Fig. 1B). The situation of single nucleotide variation (SNV) of 9 IRCR DEGs in HCC samples were summarized in Fig. 1C,D. The mutation frequency was 100% in the 9 samples. Among them, PPARGC1A, EHMT2, AIRE and PKN1 were the highest mutated genes with more than 15% mutation rates. Besides, the most common variant classification was missense mutation. In addition, we found that single nucleotide polymorphisms (SNP) play an important role in the above mutated genes, and there were six classes of base substitution and the most common class was C > T.

### Functional annotation of the IRCR DEGs

**Figure 2:**
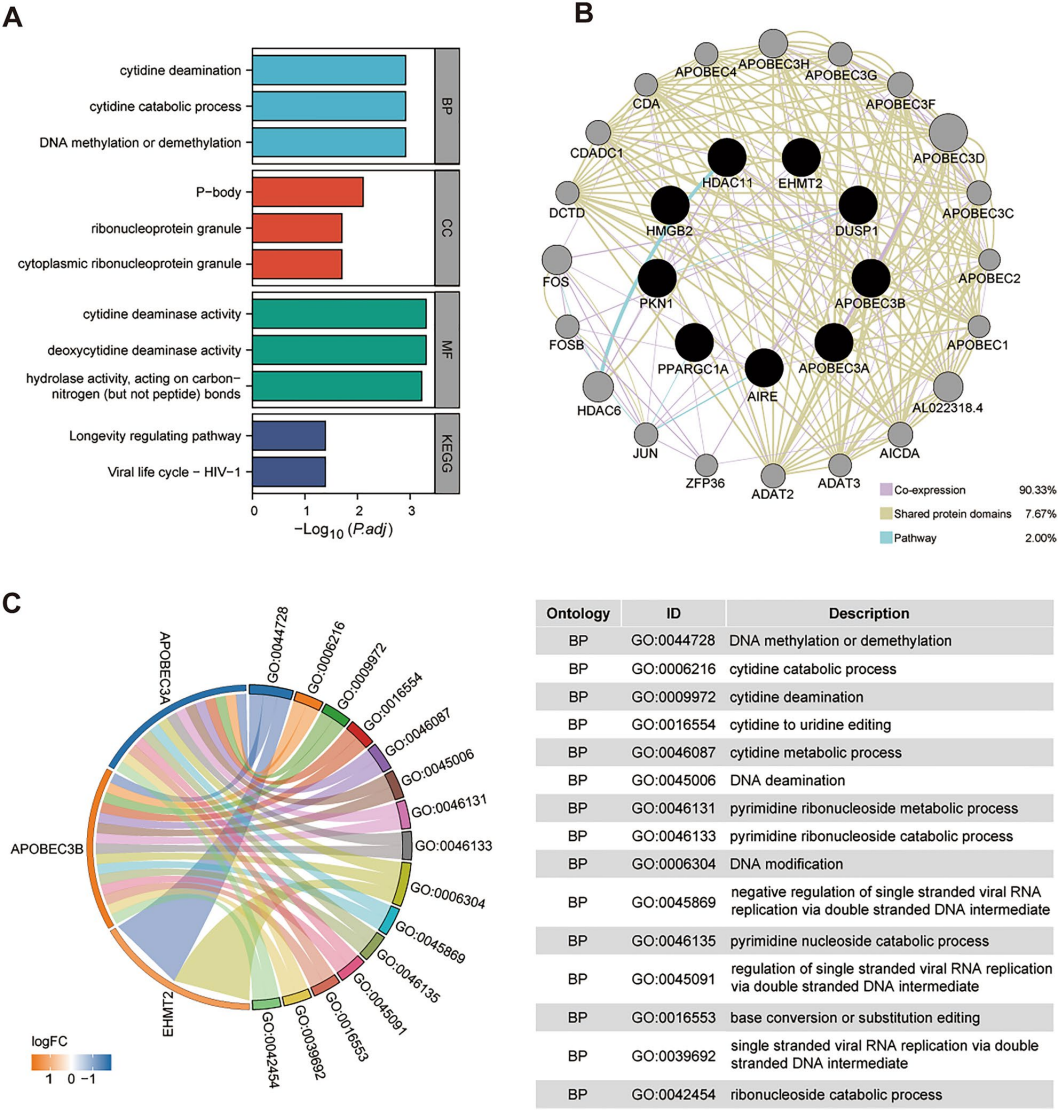
Functional annotation of IRCR DEGs. (**A**) KEGG enrichment analysis and the top 4 GO enrichment significance items of IRCR DEGs sorted by adjusted p value in BP, CC and MF. (**B**) The gene–gene interaction network of IRCR DEGs were constructed using GeneMania. (**C**) Chord plot showed the distribution of the top 15 GO enrichment in BP.

To explore the biological functions and potential mechanisms of the IRCR DEGs in the TCGA-HCC cohort, we performed GO and KEGG enrichment pathway analysis. A total of 94 Gene Ontology (GO) entries and 2 KEGG pathways were enriched in the 9 IRCRs (Supplementary Table 3). The results of biological process (BP) analysis showed that 9 IRCRs were remarkably involved in DNA methylation or demethylation, cytidine catabolic process, and cytidine deamination. Cellular component (CC) were located in P-body, cytoplasmic ribonucleoprotein granule, and ribonucleoprotein granule. Molecular function (MF) analysis suggested that cytidine deaminase activity, deoxycytidine deaminase activity, and hydrolase activity, acting on carbon–nitrogen (but not peptide) bonds were mainly enriched. From the KEGG pathway analysis, we found that these IRCRs were mainly associated with longevity regulating pathway and Viral life cycle-HIV-1 (Fig. 2A). Through the chord plot analysis of the top 15 biological processes, we found that APOBEC3A and APOBEC3B were mainly involved in the above biological processes, and revealed that they play an important role in chromatin-related biological processes (Fig. 2C).

**Figure 3:**
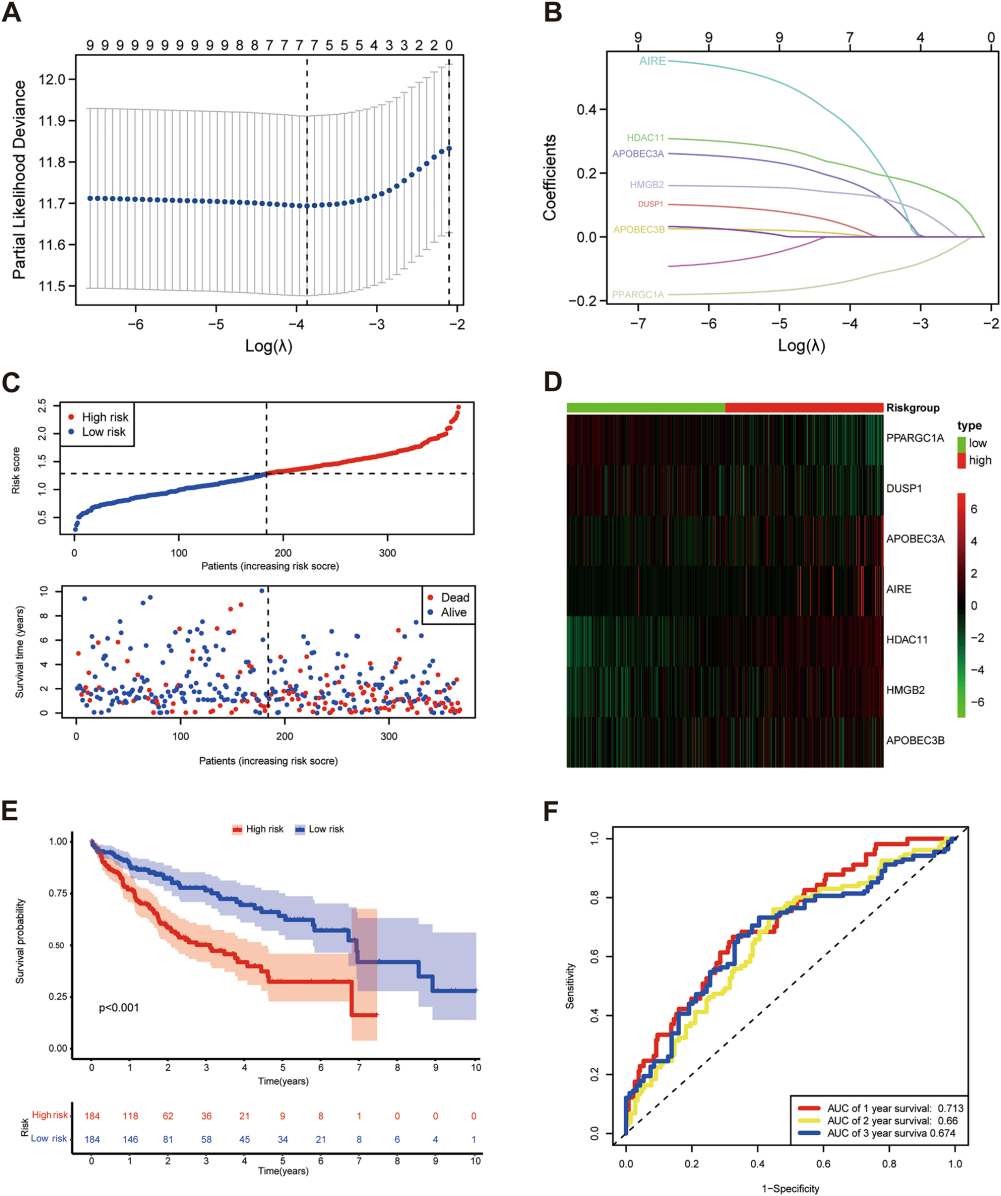
Prognostic value of risk model in HCC patients. (**A**) Ten-time cross-validation for tuning parameter selection in the LASSO model.(**B**) LASSO coefficient profiles.(**C**) Distribution of survival status based on the median risk score. (**D**) Heatmap of 7 IRCR genes in HCC patients.(**E**) Kaplan–Meier survival analysis of HCC patients between high-risk group and low-risk group. (**F**) Time-independent receiver operating characteristic (ROC) analysis of risk scores predicting 1,2,3-year overall survival.

Based on the 9 IRCR DEGs of identified and potential targets were obtained by shared protein domains, co-expression and pathway in the GeneMANIA, an entire network was constructed using Cytoscape(version 3.7.2). As shown in Fig. 3B, the results suggested that the 9 IRCR DEGs may interact with these 20 proteins, such as APOBEC1, APOBEC2, APOBEC3C, APOBEC4, ADAT2 and ADAT3, etc. (Fig. 2B).

### Construction of a prognostic model based on IRCRs

A risk model was constructed with 7 genes (PPARGC1A, DUSP1, APOBEC3A, AIRE, HDAC11, HMGB2, APOBEC3B) by using LASSO Cox regression analysis (Fig. 3A,B). The risk score was calculated by coefficients of 7 IRCRs as following formula: risk score = (-0.1335 × PPARGC1A expression) + ( 0.0302 × DUSP1 expression) + (0.1716 × APOBEC3A expression) + (0.3182 × AIRE expression) + (0.2219 × HDAC11 expression) + (0.1269 × HMGB2 expression) + (0.0219 × APOBEC3B expression) (Table 1). HCC patients were classified into two groups (high-risk group and low-risk group) according to the median risk score (Fig. 3C,D). The results of the KM curve showed that the prognosis of the high-risk group was significantly worse than that of the low-risk group (p < 0.001), which suggested that risk score was negatively correlated with prognosis (Fig. 3E). The time-dependent ROC analysis showed that the AUC values of 1, 2, and 3 years were 0.713, 0.66, and 0.674 respectively, indicating the accuracy of the model in predicting patient prognosis (Fig. 3F).

**Table 1:** Seven IRCR DEGs list and coefficient.

Gene	Coefficient
PPARGC1A	− 0.1335
DUSP1	0.0302
APOBEC3A	0.1716
AIRE	0.3182
HDAC11	0.2219
HMGB2	0.1269
APOBEC3B	0.0219

### External validation of the prognostic model

**Figure 4:**
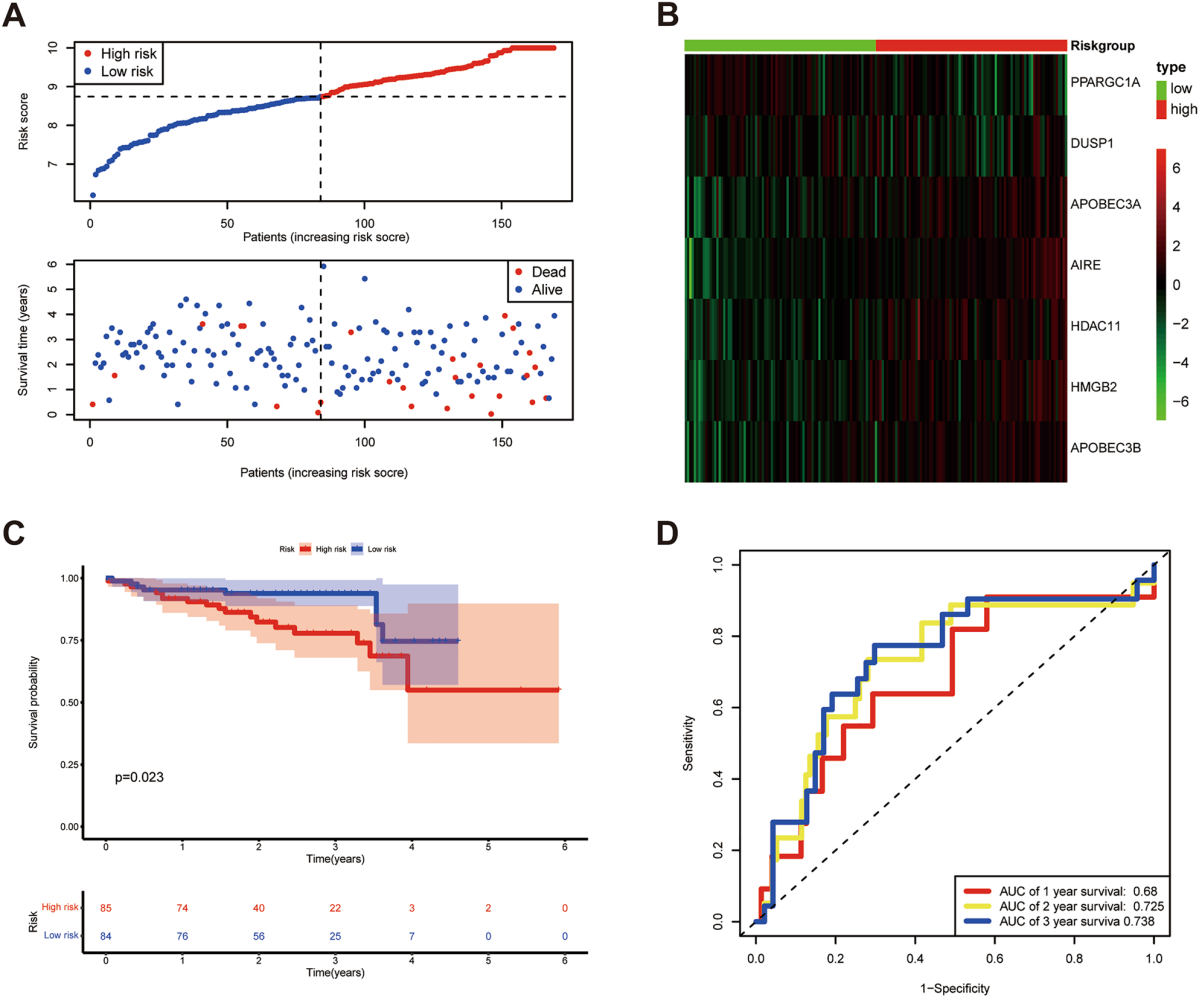
Validation of the prognostic model in ICGC cohort. (**A**) Distribution of survival status based on the median risk score. (**B**) Heatmap of 7 IRCR genes in ICGC LIRI-JP dataset. (**C**) Kaplan–Meier survival analysis of HCC patients in different risk groups. (**D**) Time-independent ROC analysis of risk scores predicting 1,2,3-year overall survival.

We divided HCC patients into low-risk and high-risk groups in the ICGC cohort based on relevant coefficients of 7 IRCRs. The results showed the distribution of survival status of each HCC patient and the heatmap of 7 IRCRs in ICGC database (Fig. 4A,B). The results of Kaplan–Meier (p = 0.023) analysis showed consistency with the TCGA cohort (Fig. 4C). In addition, the ROC curve showed AUC values of 0.713 (1 year), 0.66 (2 years) and 0.674 (3 years) (Fig. 4D).

### Correlation between the risk score and clinical characteristics

**Figure 5:**
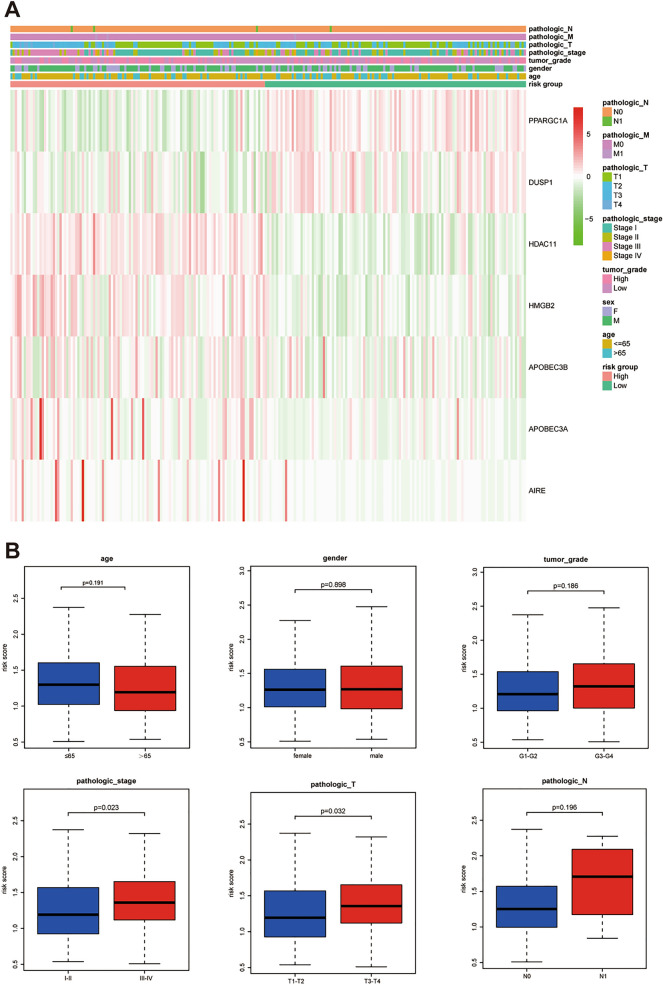
Correlation between risk score and clinical characteristics.

**Figure 6:**
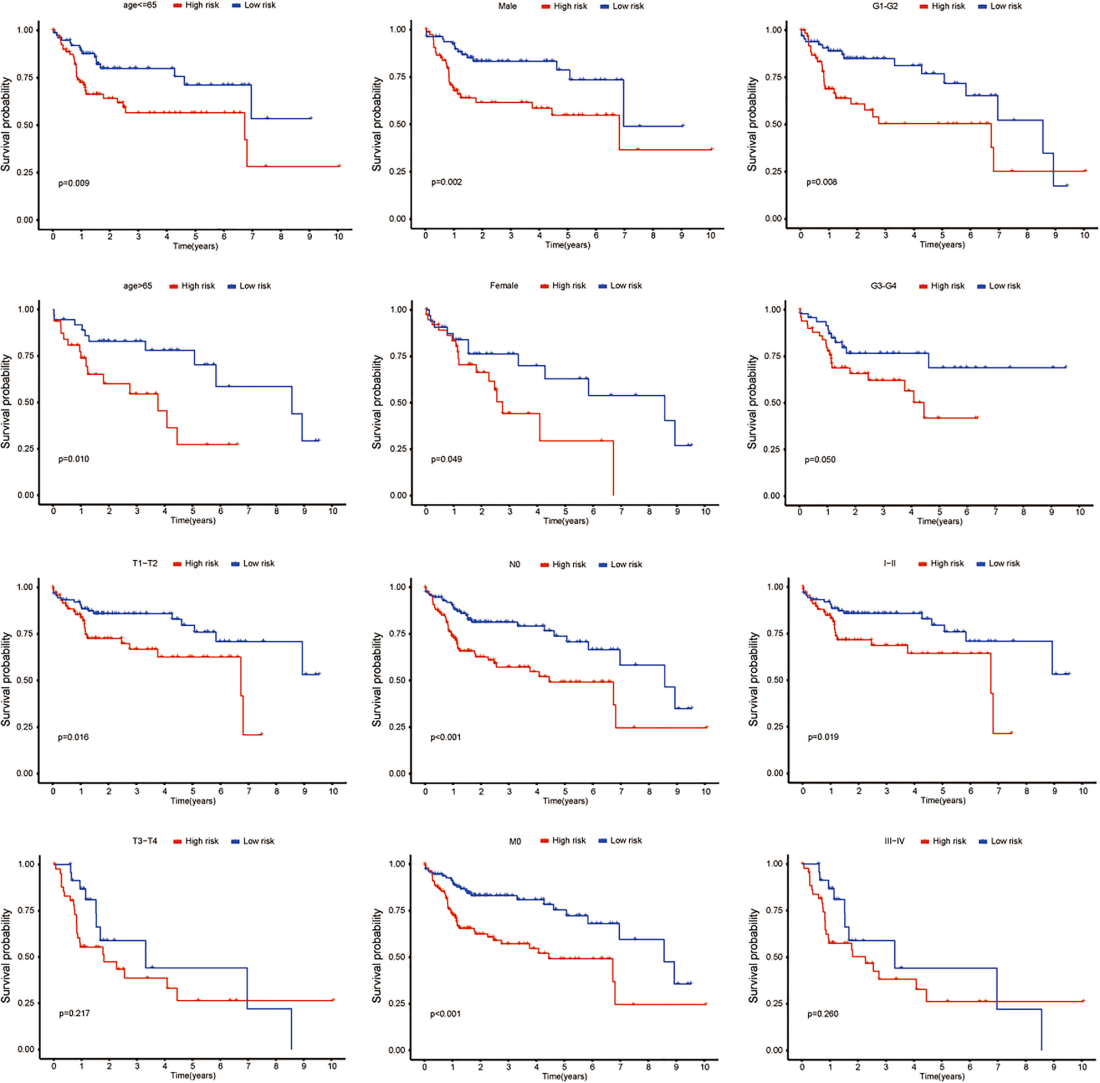
Kaplan–Meier curves of OS differences stratified by age, gender, tumor grade, N stage, T stage, M stage, or pathologic stage between the high-risk group and low-risk group.

To analyze whether the prognostic model participated in the development and progression of HCC, we used the Chi-square test to compare the risk score in different clinical characteristics. The result (Fig. 5) showed that there were significant differences between high- and low-risk groups in pathological stage (p = 0.023) and T stage (p = 0.032). Moreover, we further analyzed the prognostic significance of the signature in subgroups. The result suggested that IRCR-based signature showed excellent performance in predicting outcome in age ≤ 65 (p = 0.009), age > 65 (p = 0.010), male (p = 0.002), female (p = 0.049), G1-G2 (p = 0.008), T1-T2 stage (p = 0.016), N0 (p < 0.001), M0 (p < 0.001) and Stage I-II (p = 0.019). While IRCR-based signature showed poor performance in predicting outcome in G3-G4 (p = 0.050), T3-T4 stage (p = 0.217) and Stage III-IV (p = 0.260) (Fig. 6).

### Construction of nomogram model

**Figure 7:**
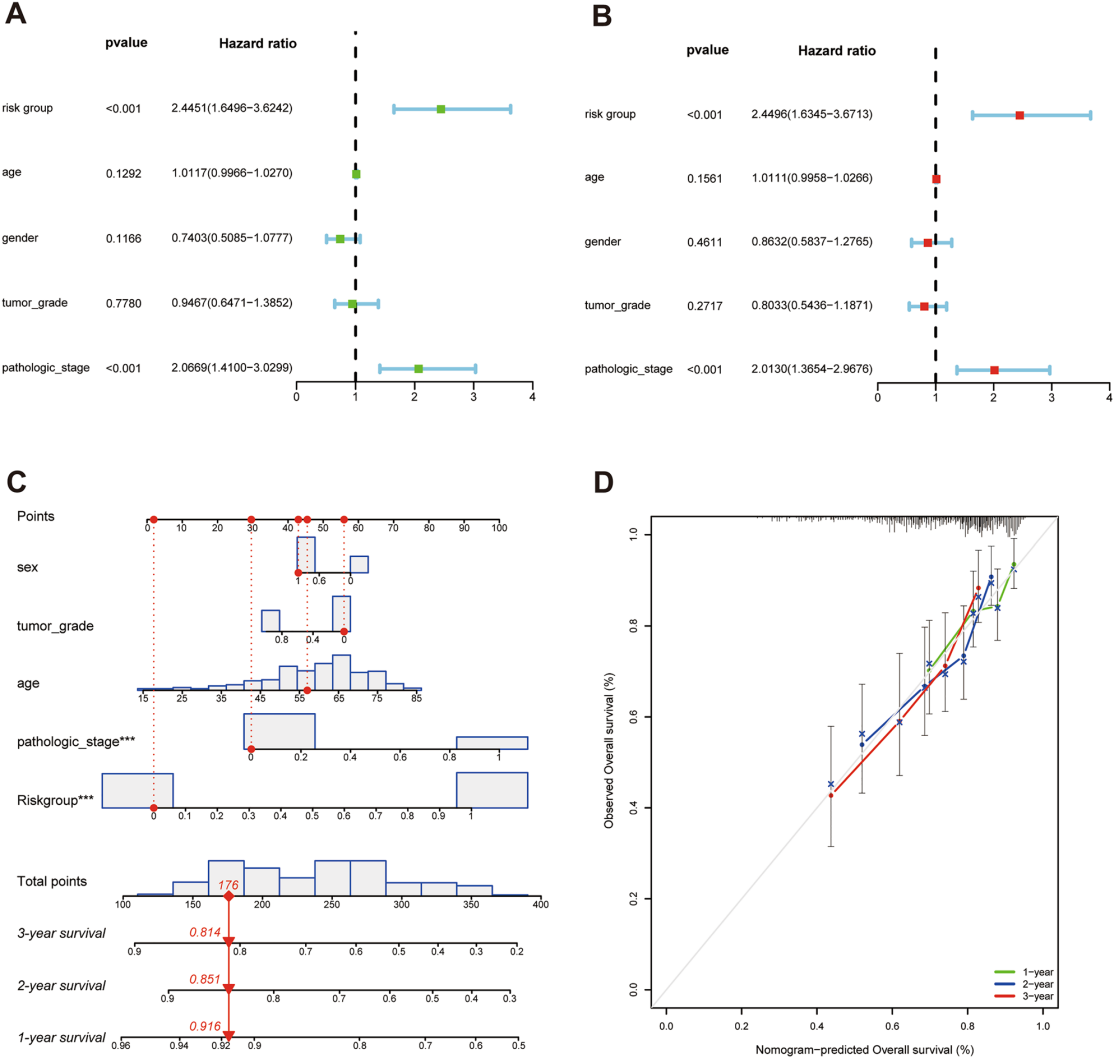
Forest plot and nomogram of the prognostic risk model. (**A**) Forest plot of univariate Cox regression analysis in HCC. (**B**) Forest plot of multivariate Cox regression analysis in HCC. (**C**)The nomogram for predicting 1-, 2-, and 3-year OS of HCC patients. (**D**) The calibration plots for predicting 1-, 2-, and 3-year OS.

Univariate COX regression analysis showed that risk group and pathologic stage were significantly relevant to the survival of HCC patients (p < 0.001) (Fig. 7A). In multivariate COX regression analysis, the risk group and pathologic stage were still remarkably related to the survival of HCC patients (p < 0.01) (Fig. 7B). Which demonstrate that IRCR-based signature was an independent prognostic factor for HCC patients.

To further forecast the survival of HCC patients, we structured a nomogram comprised of risk group, gender, tumor grade and pathologic stage. Nomography predicted the prognostic survival probability of HCC patients at 1, 2, 3 years (Fig. 7C). The calibration curve indicated that there was a good consistency between the actual survival probability and the predicted probability (Fig. 7D).

### Immune infiltration analysis of the IRCR-based signature

**Figure 8:**
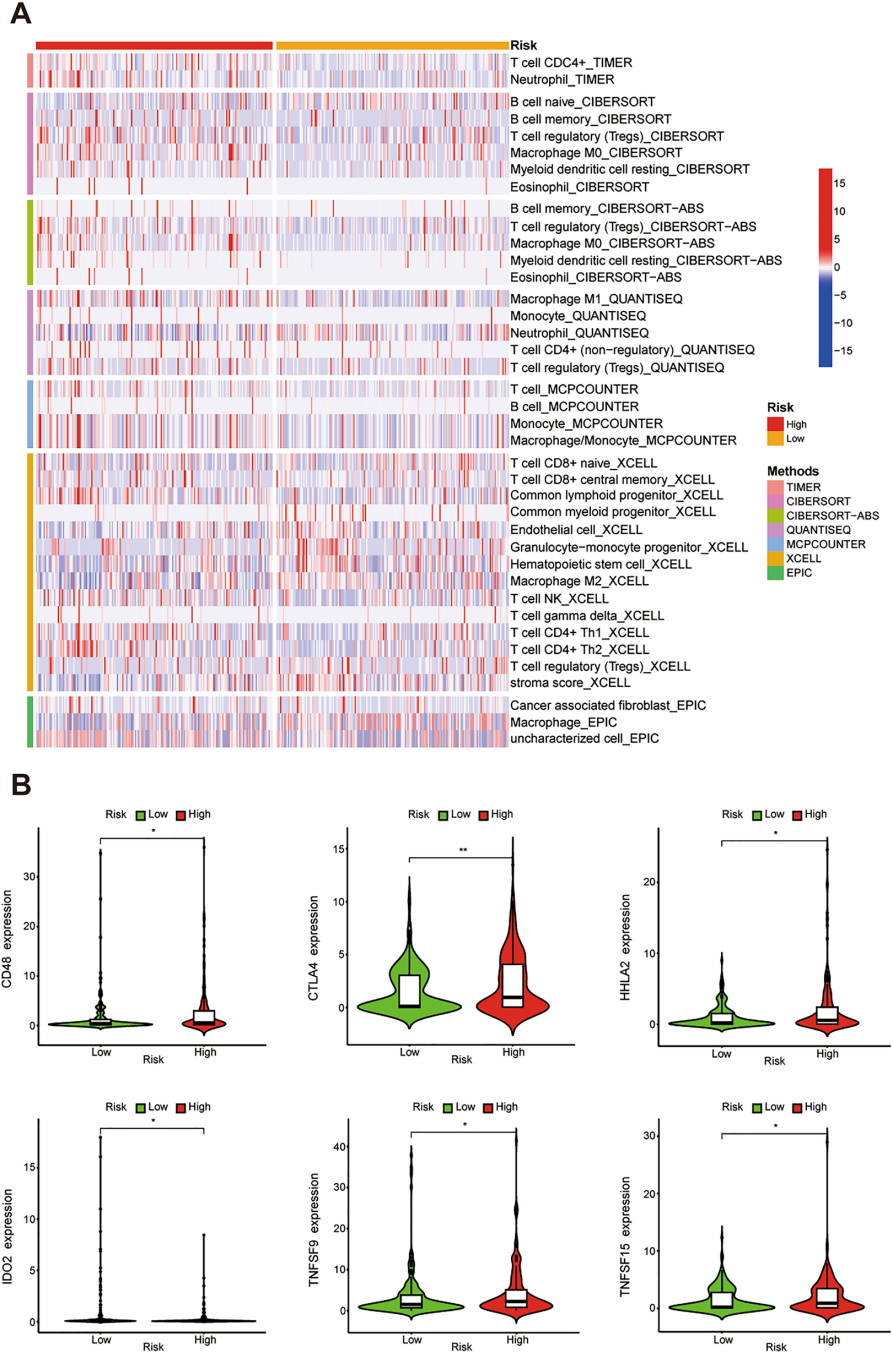
Immune infiltration and immune checkpoints analysis. (**A**) Immune cells infiltration between high-risk group and low-risk group. (**B**) The relationship between the IRCR-based signature and immune checkpoints.

According to the analyses of TIMER, CIBERSORT, CIBERSORTABS, XCELL, QUANTISEQ, EPIC, and MCP-counter, the relationship between the IRCR-based signature and immune infiltration was displayed in the heatmap (Fig. 8A). The result of XCELL indicated that the proportions of CD8 + naïve T cells, CD8 + central memory T cells, granulocyte-monocyte progenitor cells, hematopoietic stem cells, M2 macrophages, and Tregs were higher in the low-risk group, whereas myeloid dendritic cells, NK cells, Th1 cells and Th2 cells were higher in the high-risk group (Supplementary Fig. 1). We also investigated the correlation between risk groups and key immune checkpoints. The result showed that there was a difference in the expression of CD48, CTLA4, HHLA2, IDO2, TNFSF9, and TNFSF15 between the two groups. In addition, CD48, CTLA4, HHLA2, TNFSF9, and TNFSF15 were elevated in high-risk group, suggesting that the high-risk group are more likely to show immunosuppressive phenotype in tumor microenvironment (Fig. 8B).

TIMER database was used to explore the relationship between immune cells and above 7 prognostic IRCRs. The results showed HMGB2 was positively associated with all immune cells. APOBEC3A and APOBEC3B were positively associated with multiple immune cells such as B cells, CD8 + T cells, macrophage, neutrophil, and dendritic cells. HDAC11 was positively associated with B cells, CD4 + T cells, macrophage, and neutrophil. DUSP1 was positively associated with neutrophil. (Supplementary Fig. 2).

### Drug sensitivity analysis

**Figure 9:**
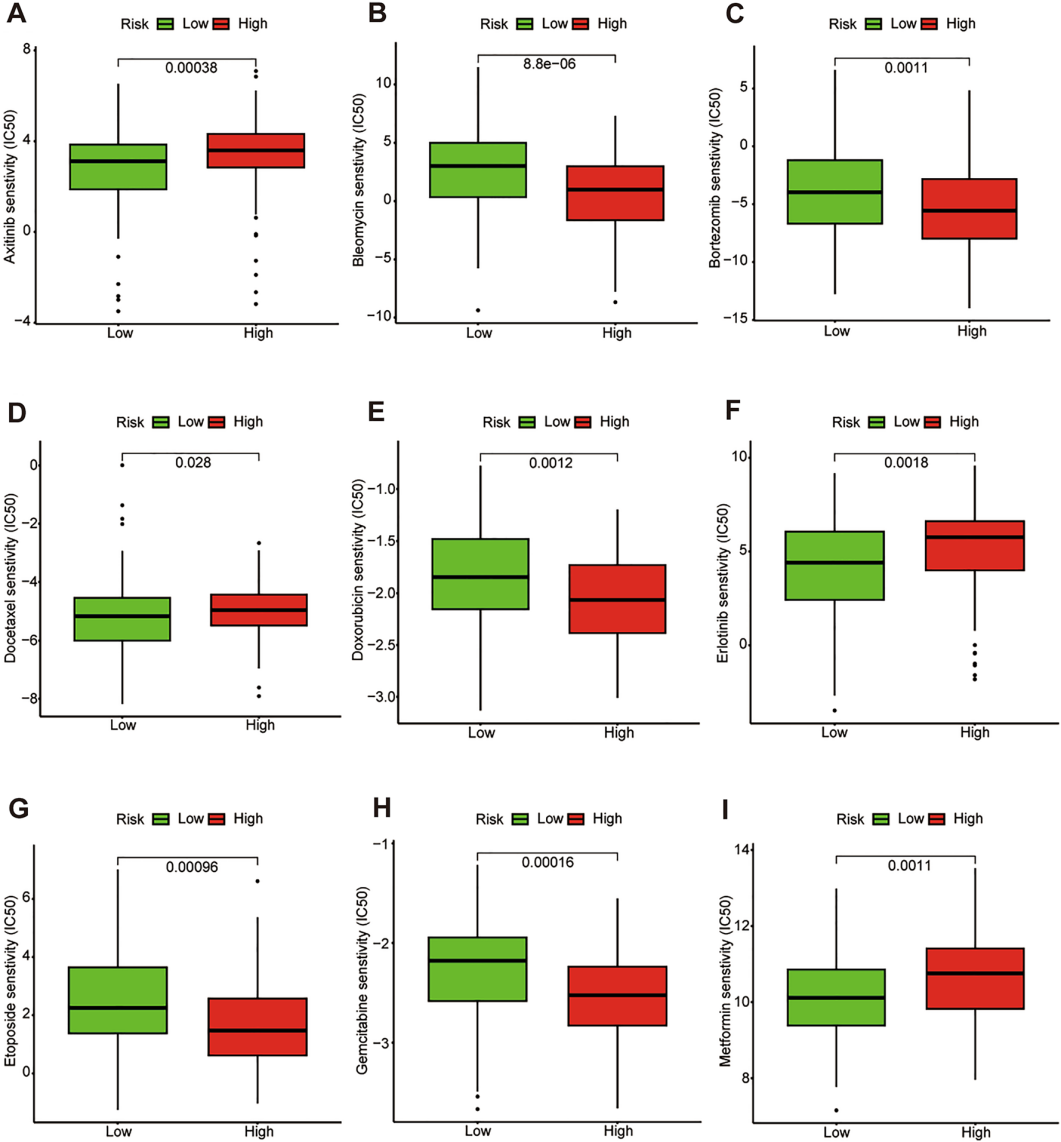
Drug sensitivity analysis.

We further investigated the differences in sensitivity of common chemotherapy drugs between the two groups in HCC patients. The results indicated that IC50 values of drugs including Axitinib, Docetaxel, Erlotinib, and Metformin were higher in the high-risk group than those of the low-risk group, which suggested that HCC patients in the high-risk group were much more sensitive to these drugs (Fig. 9). While IC50 values of Bleomycin, Bortezomib, Doxorubicin, Etoposide, and Gemcitabine were significantly lower in the low-risk group than those of the high-risk group, suggesting that HCC patients in the low-risk group were much more sensitive to these drugs.

### Comparison with other risk prognostic models in HCC

**Figure 10:**
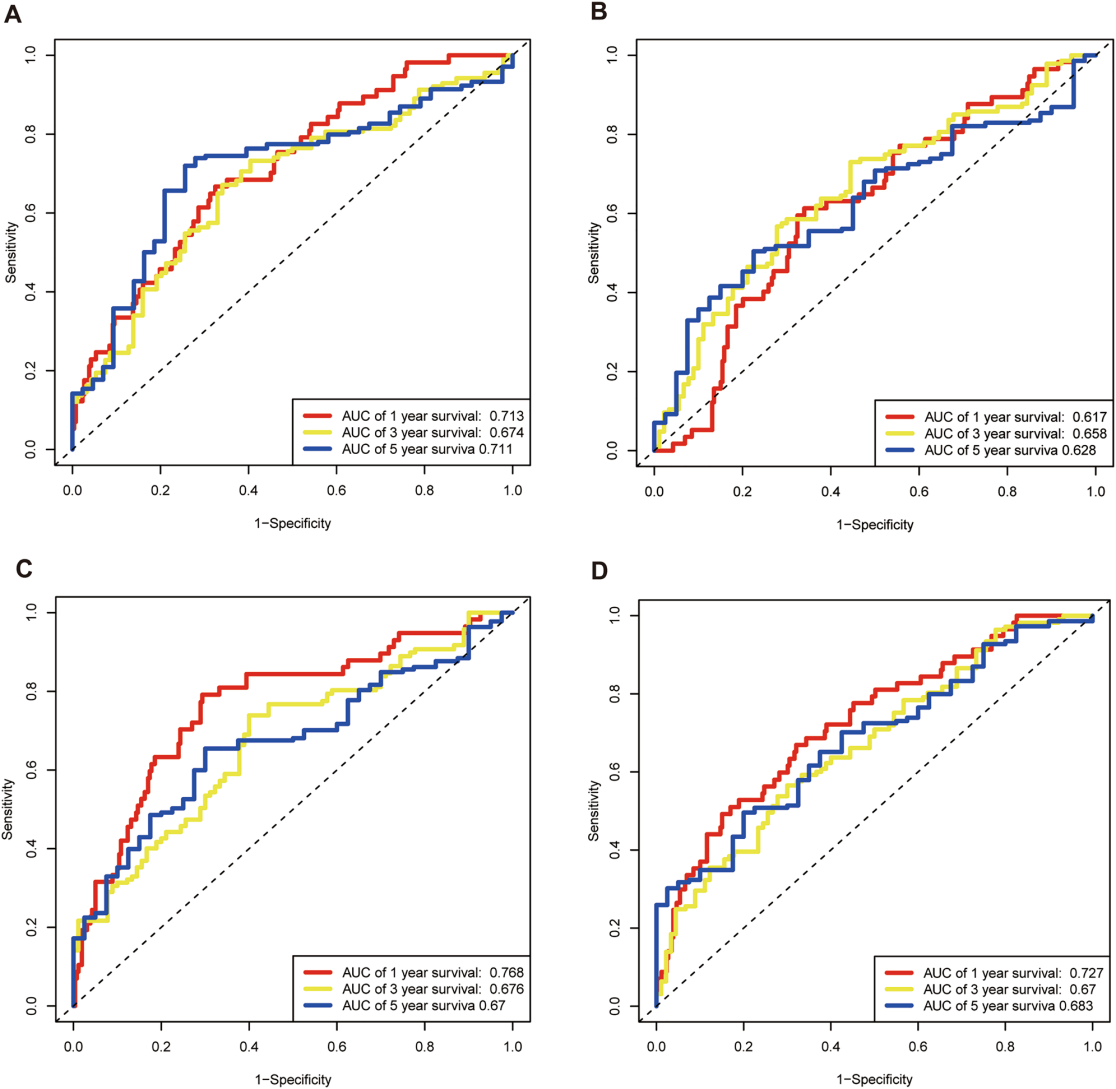
The comparison of our prognostic model and other models. (**A**) Time-dependent ROC analysis for our prognostic model. (**B**) Time-dependent ROC analysis for four-gene model. (**C**) Time-dependent ROC analysis for seven-CRs model. (**D**) Time-dependent ROC analysis for the four-immune-related gene model.

To evaluate the prognostic ability of our model for HCC, we compared other three prognostic models: the four-gene model^43^, the seven-CRs model^44^, and the four-immune-related-gene model^45^. For the TCGA-LIHC dataset, we used methods of externally validating our model to calculate the corresponding risk scores. The results showed that the AUC values for 1,3, and 5-year survival of the four-gene model were lower than our model, the AUC values for 1-year survival of the seven-CRs model and four-immune-related-gene model were slightly higher than our model, but the AUC values for five-year survival were lower (Fig. 10). These results suggested that our model was advantageous in predicting the long-term survival (5-year) of HCC patients.

## Discussion

There are about 906,000 new cases and 830,000 deaths of primary liver cancer worldwide in 2020, severely threaten human health and life^46^. Hepatocellular carcinoma (HCC) is still the most common pathological type. Cancer immunotherapies has greatly changed the clinical treatment of HCC in recent years, but it remains one of the worst prognosis diseases due to the heterogeneity. Epigenetic alterations can influence the interactions between tumor cells and liver tumor microenvironment (TME), so the epigenetics study can enhance anti-tumor immunity and better combat HCC^47^. A growing number of studies have shown that CRs plays an important role in HCC. Therefore, the analysis of HCC sequencing data by combining CRs and immune-related genes could be beneficial in the search for new biomarkers to predict the response to immunotherapy, and provide potential therapeutic targets for the treatment of HCC.

In this study, we innovatively analysed CRs with immune-related genes in HCC, established a risk model associated with 7 IRCR and verified in ICGC LIRI-JP cohort. Univariate and multivariate COX analysis showed that the risk score based on 7 IRCR was an independent prognostic indicator for HCC patients. Compared with other prognostic models, our model is advantages in predicting long-term survival of HCC patients. In addition, we also analysed the relationship between the signature and immune cells infiltration in HCC.

GO analysis showed that IRCRs were mainly related to biological processes (BP), such as DNA methylation or demethylation, cytidine catabolic process, cytidine deamination, cytidine to uridine editing, and cytidine metabolic process. The result of KEGG pathway enrichment analyses indicated that IRCRs were mainly involved in the longevity regulating pathway and viral life cycle-HIV-1. Aging was a universal feature of organisms and tumorigenesis was also closely associated with cellular senescence, including HCC, and was regulated by longevity signaling pathways^48,49^. APOBEC3A and APOBEC3B were involved in most biological processes, and the relationship between the APOBEC (apolipoprotein B mRNA editing enzyme, catalytic polypeptide-like) family members was the most significant according to the gene- gene interaction network. APOBEC family was the main source of DNA modification of cancer genome, participated in immune response and antiviral response in human body with specific mutation pattern^50^.

Among the APOBEC protein family members, APOBEC3A and APOBEC3B are able to restrict the infection of multiple viruses, including parvovirus, hepatitis B virus (HBV), human papillomavirus, human immunodeficiency virus 1 (HIV-1) and carcinogenesis^51–54^. HBV is the main risk factor for HCC, but epigenetic factors are also involved in the underlying pathogenesis of HCC. APOBEC3A is an editing molecule of HBV DNA, APOBEC3A and APOBEC3B play crucial roles in inducing HBV DNA degradation^55^. Duowei found that APOBEC3B increased transcriptional expression through the non-classical NF-κB signal pathway, while the increased expression of APOBEC3B significantly increased CCL2 chemokine, thus recruiting myeloid-derived suppressor cells (MDSCs) and tumor-associated macrophages (TAMs) to participate in the development of HCC^56^. HMGB2, as a member of high-mobility group box(HMGB) proteins family, is involved in DNA replication, repair, transcription, differentiation, proliferation, cell signaling, inflammation, tumor migration, and cellular senescence^57,58^. It has been reported that HMGB2 gene knockout can induce cell senescence and inhibit the growth of tumor cells^59^. Cyclic cGMP-AMP synthase (cGAS) promotes inflammatory senescence-associated secretory phenotype (SASP) by recognizing cytoplasmic chromatin during cellular senescence. HMGB2 can retain the function of topoisomerase 1-DNA covalent cleavage complex (TOP1cc) in cytoplasmic chromatin. HMGB2-TOP1cc-cGAS axis functionally regulates SASP and immune checkpoint blocking response^60^. Dual-specificity protein phosphatase 1(DUSP1) expression declined in HCC tissue and was significantly associated with HCC progression and aggressiveness. DUSP1 down-regulation depends on promoter hypermethylation associated with loss of heterozygosity or ERK/SKP2/CKS1-dependent ubiquitination^61^. Hao revealed that DUSP1 expression correlated with the activation of p53, which in turn positively regulated DUSP1 transcription. If this destruction of the positive regulatory loop could contribute to HCC development and progress^62^. Autoimmune regulatory factor (AIRE) was a transcription factor mainly expressed in thymic medulla epithelial cells. AIRE expression was also found in other tissues outside the thymus^63^. Zhu showed that AIRE deficiency in mice led to increased immune response to melanoma and increased infiltration of CD4 + and CD8 + in tumor tissue, spleen and tumor draining lymph nodes^64^. HDAC11 was the sole class IV member of the histone deacetylases (HDAC) family and the smallest HDAC enzyme identified to date^65^. HDAC11 induced deacetylation of p53 transcription factor Egr-1 (early growth response 1), which prevented p53 transcription and promoted the development of HCC^66^. In a murine model, T cells lacking HDAC11 showed proinflammatory cytokine production and effector molecule expression^67^. PPARGC1A (peroxisome proliferator activated receptor gamma coactivator 1 alpha, PGC-1 α) was a transcriptional coactivator with important roles in mitochondrial biosynthesis, homeostasis, and energy metabolism^68^. PPARGC1A had oncogenic and tumor suppressive features, and high and low levels of PPARGC1A expression associated with the prognosis of different cancers. Compared with normal liver tissue, the expression of PPARGC1A in HCC tissue is downregulated and acted as a tumor inhibitory role in the occurrence and development of HCC^69^. Additionally, TIMER database showed that 7 IRCRs of the model were related to immune cells, which revealed that IRCRs might regulate HCC progression by influencing immune infiltration.

There were significant differences in immunotherapy among different patients, which was caused by the heterogeneity of immune environment in tumor microenvironment. Our results showed that the proportion of CD8 + T cells were higher in low-risk group, and the expressions of CD48, CTLA4, HHLA2, TNFSF9 and TNFSF15 in high-risk group were higher than those in the low-risk group, which suggested that the poor prognosis of HCC patients in high-risk group might be related to the immunosuppressive microenvironment. Moreover, HCC patients in high-risk group might benefit from checkpoint inhibitor immunotherapy. Also, we found that HCC patients in high-risk group might benefit from the treatments of Axitinib, Docetaxel, Erlotinib, and Metformin, while HCC patients in low-risk group might benefit from the treatments with Bleomycin, Bortezomib, Doxorubicin, Etoposide, and Gemcitabine.

Although the study could provide excellent aid for accurately treating HCC patients, there are still some shortcomings. First, although there is a large amount of high-throughput data stored in the TCGA database, the number of samples related to HCC is still insufficient. Second, the above results need to be further verified in vitro and in vivo. Therefore, further research is necessary to address the possible limitations in terms of results and conclusions.

## Conclusion

We identified differentially expressed IRCRs and found that IRCRs are important for predicting the prognosis of HCC patients, and targeting IRCRs could be expected as an effective treatment for HCC. In conclusion, our prognostic model could provide new ideas and research directions for the accurate treatment of HCC.

### Supplementary Information

Supplementary Tables.

Supplementary Figure 1.

Supplementary Figure 2.

## Data Availability

The datasets generated analysed during the current study are available in the UCSC Xena repository, we can download liver cancer related RNA sequencing and phenotype data from the link below, https://xenabrowser.net/datapages/?cohort=GDC%20TCGA%20Liver%20Cancer%20(LIHC)&removeHub=https%3A%2F%2Fxena.treehouse.gi.ucsc.edu%3A443.
