# Supplementary figures and images for: Seven chromatin regulators as immune cell infiltration characteristics, potential diagnostic biomarkers and drugs prediction in hepatocellular carcinoma
Source: Sci Rep. 2023 Oct 30;13:18643. doi: 10.1038/s41598-023-46107-x (PMC10616163; doi:10.1038/s41598-023-46107-x)

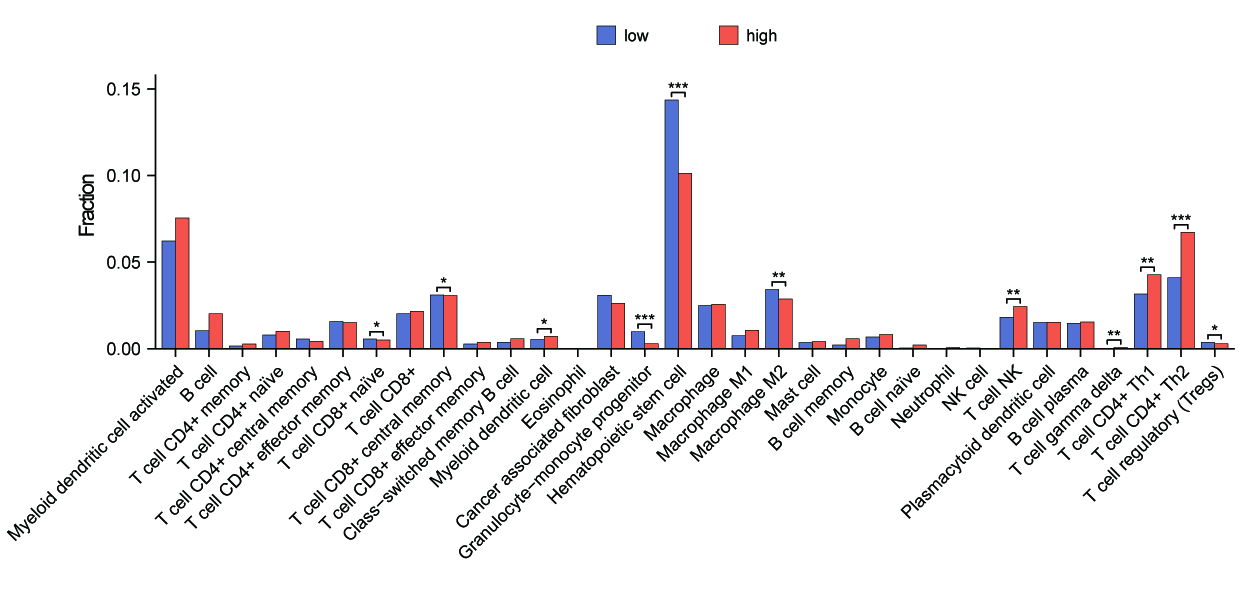

Supplement: Supplementary file 2 — Supplementary Figure 1. [file 41598_2023_46107_MOESM2_ESM.tif]

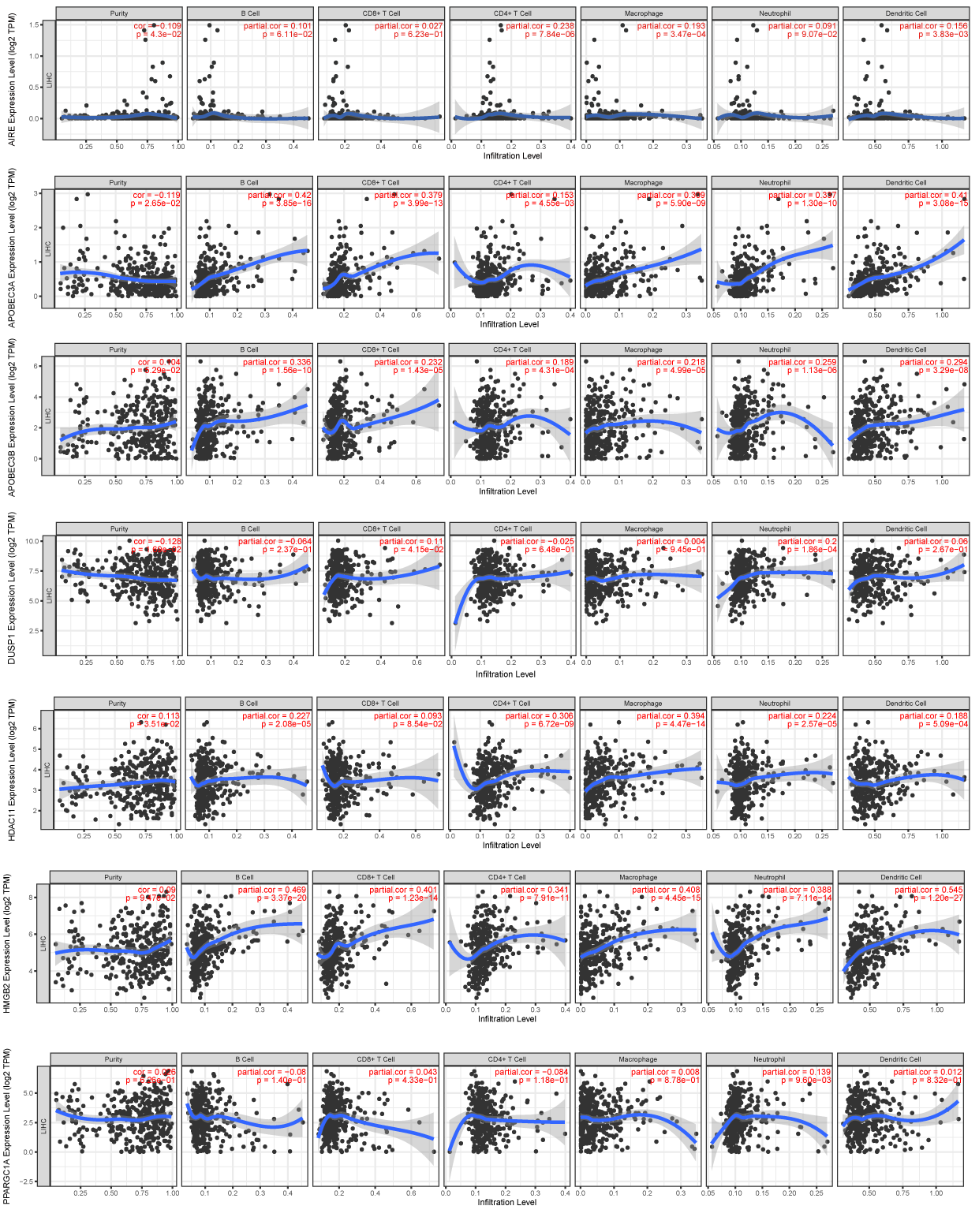

Supplement: Supplementary file 3 — Supplementary Figure 2. [file 41598_2023_46107_MOESM3_ESM.tif]
